# Prevalence and Influencing Factors of Comorbid Major Depressive Disorder in Elderly Schizophrenia: A Multicenter Cross-Sectional Study

**DOI:** 10.31083/AP52709

**Published:** 2026-09-07

**Authors:** Peng Cui, Guoshuai Luo, Xiangyang Zhang

**Affiliations:** ^1^Institute of Mental Health, Tianjin Anding Hospital, Psychiatric Medical Center of Tianjin University, Mental Health Center of Tianjin Medical University, 300222 Tianjin, China; ^2^Hefei Fourth People’s Hospital, Anhui Mental Health Center, Affiliated Mental Health Center, Anhui Medical University, 230022 Hefei, Anhui, China

**Keywords:** schizophrenia, major depressive disorder, comorbidity, PANSS, geriatric psychiatry

## Abstract

**Background:**

This multicenter cross-sectional study aimed to determine the prevalence, clinical correlates, and determinants of comorbid major depressive disorder (MDD) in elderly patients with schizophrenia (SZ), addressing a critical gap in understanding late-life dual diagnosis.

**Methods:**

We recruited 274 elderly SZ patients (≥60 years) from nine psychiatric centers across China. Diagnoses of schizophrenia and concurrent MDD were confirmed using the Structured Clinical Interview for DSM-5 (SCID-5). Depressive severity was assessed with the Hamilton Depression Rating Scale (HAMD-17), and psychotic symptoms with the Positive and Negative Syndrome Scale (PANSS). Multivariate analyses (binary/ordinal logistic regression, partial correlations), adjusted for age, sex, education, and antipsychotic dosage (chlorpromazine equivalents), identified factors associated with MDD comorbidity and severity.

**Results:**

The prevalence of comorbid MDD was 29.2% (80/274). PANSS General Psychopathology subscale scores (odds ratio [OR] = 1.064, 95% confidence interval [CI]: 1.013–1.118, *p* = 0.012) and PANSS Total Score (OR = 1.062, 95% CI: 1.008–1.152, *p* = 0.002) were significantly associated with MDD comorbidity. HAMD-17 scores exhibited strong positive correlations with PANSS General Psychopathology (r = 0.402, *p* < 0.001) and PANSS Total Score (r = 0.310, *p* < 0.001) after adjustment for covariates. No significant association was found between HAMD-17 and the PANSS Negative subscale (r = 0.606, *p* > 0.05).

**Conclusions:**

In this multicenter sample of elderly Chinese inpatients with schizophrenia, nearly one-third of participants had comorbid MDD. The robust association between overall psychopathology severity, as measured by the PANSS Total Score and General Psychopathology subscale, and comorbid MDD supports the presence of transdiagnostic mechanisms in late-life schizophrenia. PANSS assessment, particularly the General Psychopathology subscale, may serve as a clinically informative adjunct to routine evaluation for identifying elderly patients with schizophrenia who are at increased risk of comorbid depression. However, its modest discriminative performance (area under the curve [AUC] ≈ 0.60) precludes its use as a standalone screening tool*.*

## Main Points

1. In elderly schizophrenia, the prevalence of comorbid major depressive disorder (MDD) was 29.2%, with Positive and Negative Syndrome Scale (PANSS) general psychopathology and total scores independently associated.

2. Hamilton Depression Rating Scale (HAMD‑17) scores correlated with PANSS general and total, but not negative, subscales after covariate adjustment.

## 1. Introduction

Schizophrenia (SZ) is a severe mental disorder often complicated by comorbid major depressive disorder (MDD); a combination associated with significant clinical and functional impairments [1,2]. The recent meta-analysis revealed a pooled prevalence of 32.6% for MDD in stabilized SZ outpatients, underscoring its high burden [1]. Notably, this comorbidity is linked to poorer outcomes, including increased suicidality, weight gain, and reduced quality of life [3,4]. Despite its prevalence, schizophrenia comorbid with major depressive disorder (SZ-MDD) remains underdiagnosed and undertreated, with only half of affected patients receiving antidepressants and fewer achieving remission [3].

While historically classified as “non-affective”, depressive pathology is now recognized as a highly prevalent comorbidity, with an incidence rate of 34.4% to 80% in patients with first-episode schizophrenia [5,6]. However, the heterogeneity in reported prevalence rates (16–69%) highlights methodological challenges, particularly variations in assessment tools [1,7]. Studies using self-reported questionnaires (e.g., Patient Health Questionnaire [PHQ]) report higher rates (46.9%) than clinician-rated scales (e.g., the Calgary Depression Scale for Schizophrenia [CDSS]; 30.1%) or structured interviews (16.4%) [1]. This discrepancy suggests potential overestimation in self-reports or under-detection by clinicians, underscoring the need for standardized diagnostic approaches [1].

While existing research has identified moderators of SZ-MDD, such as older age, lower education, substance use, and chronic somatic illnesses [1], most studies focus on younger or middle-aged populations [8,9,10]. Older adults with SZ-MDD represent a critically understudied group, despite facing unique challenges like polypharmacy, cognitive decline, and social isolation [11]. Neurobiological aging may fundamentally alter depressive phenotypes—blurring boundaries between apathy, negative symptoms, and dementia prodromes—while cultural factors (e.g., somatic symptom reporting biases) further complicate assessment [12]. Older adults may also exhibit distinct depressive phenotypes due to neurodegenerative changes or cumulative psychosocial stressors. Yet, no studies have examined whether the high comorbidity rates and influencing factors observed in younger SZ-MDD patients [10,13,14] persist in the elderly demographic.

Despite this high prevalence and severe impact, depressive pathology remains frequently overlooked in clinical management, partly due to diagnostic overshadowing by positive/negative symptoms and nosological uncertainties [15,16,17]. Furthermore, the geriatric SZ-MDD cohort likely faces an exacerbated burden due to age-related vulnerabilities such as multimorbidity, polypharmacy, and social isolation [18,19], necessitating research to delineate their distinct clinical profile. The current study aims to address these gaps by investigating the prevalence, clinical correlates, and diagnostic challenges of MDD in elderly SZ patients, leveraging multicenter data from China. By elucidating age-specific features and moderators, we seek to inform targeted interventions for this vulnerable population.

## 2. Materials and Methods

### 2.1 Ethics Approval and Participants

This study was approved by the Ethics Committee of the Institute of Psychology of the Chinese Academy of Sciences; all participants provided written informed consent. We enrolled 274 elderly schizophrenia patients (aged ≥60 years per World Health Organization [WHO] criteria) [20,21] from nine psychiatric services (Tianjin Anding Hospital, Guangzhou Huiai Hospital, Hebei Provincial Veterans Hospital, Wenzhou Kangning Hospital, Shanghai Putuo District Mental Health Center, Changshu Third People’s Hospital, Shenyang Anning Hospital, Wuhan Mental Health Center, Shanghai Pudong New District Mental Health Center) during 2022–2023. All centers are tertiary psychiatric hospitals affiliated with regional mental health networks. Patients were identified through inpatient admission records, and those meeting eligibility criteria were approached for participation by trained research coordinators at each site. Enrollment was consecutive to minimize selection bias.

Diagnoses were confirmed using the Structured Clinical Interview for DSM-5 (SCID-5), requiring concurrent fulfillment of schizophrenia and major depressive episode criteria. Inclusion permitted chronic comorbidities (hypertension, type 2 diabetes, coronary heart disease); exclusions were acute/severe conditions (e.g., acute cerebral hemorrhage/infarction, coma, myocardial infarction, renal failure).

The study recruited participants from nine psychiatric centers across China, with site-wise sample sizes ranging from 22 to 38 patients per center. All participants were recruited from inpatient settings. All clinical assessments (SCID-5, Hamilton Depression Rating Scale [HAMD-17], and Positive and Negative Syndrome Scale [PANSS]) were conducted by trained psychiatrists or clinical psychologists at each site. Prior to study initiation, all raters participated in a centralized training workshop, which included didactic lectures, video-based case vignettes, and live patient interviews. After training, each rater was required to achieve an intraclass correlation coefficient (ICC) ≥0.80 for each scale against gold-standard ratings from the coordinating center (Tianjin Anding Hospital). During the study period, inter-rater reliability was maintained through quarterly calibration meetings and random cross-site review of 10% of audio-recorded interviews. The ICCs for the study period were: HAMD-17 = 0.89 (95% confidence interval [CI]: 0.85–0.93), PANSS total = 0.91 (95% CI: 0.88–0.94), and SCID-5 diagnostic agreement = 94.2% (κ = 0.88).

### 2.2 Clinical Assessments

Assessments included: (1) HAMD-17: Evaluated depressive severity (scores: ≤7 = no depression; 8–16 = mild; 17–23 = moderate; ≥24 = severe) [8]; (2) PANSS: Assessed psychotic symptoms (Positive Syndrome, Negative Syndrome, General Psychopathology Syndrome subscales) [22]; (3) Demographics/clinical variables: age, sex, education years, marriage status, family psychiatric history, age of first-onset, body mass index (BMI), and chlorpromazine equivalent dose.

### 2.3 Statistical Analysis

Analyses used SPSS 25.0 (IBM Corporation, Armonk, NY, USA; α = 0.05, two-tailed). Kolmogorov-Smirnov tests assessed normality; group comparisons employed *t*-tests/one-way analysis of variance (ANOVA) (continuous) or χ^2^ tests (categorical). Binary logistic regression identified MDD comorbidity-associated factors (dependent: MDD yes/no; covariates: age, sex, antipsychotic dose). Receiver operating characteristic (ROC) analysis evaluated discriminative capacity (area under the curve [AUC] >0.7 = acceptable). Ordinal logistic regression analyzed associated factors of depression severity (dependent: 0 = no, 1 = mild, 2 = moderate, 3 = severe depression). HAMD-PANSS associations used: partial correlation (adjusted for age, sex, education years, age of first-onset); multivariate regression (HAMD-17 dependent, PANSS subscales independent; covariates: age, sex, education, onset age). Bonferroni correction addressed multiple comparisons. The ROC analysis for HAMD-17 was included as a positive-control/construct-overlap validation to confirm the robustness of depression ascertainment using SCID-5, rather than as a primary predictive analysis. The PANSS General Psychopathology (AUC = 0.598) and PANSS total score (AUC = 0.595) showed only modest discriminative ability, indicating that these indices should not be interpreted as standalone screening tools but rather as correlates of MDD comorbidity.

To account for potential site-level clustering, we additionally fitted generalized linear mixed models (GLMM) with site as a random intercept, while retaining the same fixed covariates as in the primary logistic regression models. The results were consistent with those from the standard models (site-level variance minimal, ICC <0.05), indicating that center-level heterogeneity did not substantially influence the findings. For simplicity and interpretability, we present the conventional regression results in the main text, with the GLMM results noted as confirmatory. The proportional odds assumption for ordinal logistic regression was tested using the test of parallel lines and was satisfied (*p* = 0.312).

All multivariate regression models (binary logistic regression for MDD comorbidity and ordinal logistic regression for depression severity) were adjusted for a prespecified core set of covariates: age, sex, education years, BMI, chlorpromazine equivalent dose, diabetes mellitus, and hypertension. For partial correlation analyses examining HAMD-PANSS associations, adjustments were made for age, sex, education years, age of first-onset, and chlorpromazine equivalent dose; BMI and chronic somatic conditions were not included as covariates in partial correlation analyses, as this method is designed for continuous variables and inclusion of binary covariates is not standard practice.

## 3. Results

### 3.1 Prevalence of Major Depressive Disorder in Elderly Schizophrenia Patients

A total of 274 elderly schizophrenia patients (mean age 64.26 ± 3.08 years; 198 males, 76 females) were enrolled. The mean age of first onset was 29.71 ± 10.33 years, and the mean education duration was 8.60 ± 2.62 years. The prevalence of SZ-MDD was 29.2% (80/274). The HAMD-17 total score averaged 8.07 ± 6.29. The PANSS scores were: Positive Syndrome 15.57 ± 5.49, Negative Syndrome 23.76 ± 6.72, General Psychopathology Syndrome 39.74 ± 8.92, and total score 79.07 ± 16.69 (Table 1).

**Table 1.** **Demographic and clinical characteristics of elderly patients with schizophrenia**.

Variables		Frequency/Mean	%/SD
Gender			
	Male	198	72.3
	Female	76	27.7
SZ-MDD			
	No	194	70.8
	Yes	80	29.2
Marriage status			
	No-Married	152	55.7
	Married	79	28.9
	Divorced	32	11.7
	Widowed	10	3.7
Family history			
	No	247	90.1
	Yes	27	9.9
Diabetes mellitus			
	No	196	71.5
	Yes	78	28.5
Hypertension			
	No	199	72.6
	Yes	75	27.4
Cardiovascular disease			
	No	255	94.8
	Yes	14	5.2
Age (years)		64.26	3.08
Education years (years)		8.60	2.62
Age of first onset (years)		29.71	10.33
BMI (kg/m^2^)		23.96	5.03
Chlorpromazine equivalent dose		418.39	233.01
	Positive Syndrome	15.57	5.49
	Negative Syndrome	23.76	6.72
	General Psychopathology Syndrome	39.74	8.92
PANSS total score		79.07	16.69
HAMD total score		8.07	6.29
	No depression	77	28.1
	Mild depression	103	37.6
	Moderate depression	41	15.0
	Severe Depression	53	19.3

Note: SZ-MDD, schizophrenia with comorbid major depressive disorder; BMI, body mass index; PANSS, Positive and Negative Syndrome Scale; HAMD, Hamilton Depression Rating Scale.

### 3.2 Characteristics Comparison by MDD Comorbidity Status

Based on HAMD-17 severity, 28.1% (77/274) were non-depressed, 37.6% (103/274) had mild depression, 15.0% (41/274) had moderate depression, and 19.3% (53/274) had severe depression (Table 1).

SZ-MDD patients had more years of education than non-SZ-MDD patients (9.15 ± 2.93 vs. 8.37 ± 2.44, *p* = 0.026). After age-sex adjustment, the chlorpromazine equivalent dose was higher in the SZ-MDD group (486.93 ± 209.98 vs. 252.99 ± 162.70; B = 0.85, Wald χ^2^ = 23.39, *p* < 0.0001; OR = 3.33, 95% CI: 1.29–5.71). SZ-MDD patients showed higher HAMD-17, PANSS Positive Syndrome, General Psychopathology Syndrome, and total scores (all *p* < 0.05). No differences in age, age of first-onset, BMI, sex distribution, family history of mental illness, diabetes mellitus, hypertension, or cardiovascular disease (all *p* > 0.05, Table 2).

**Table 2.** **Comparison of characteristics between elderly patients with schizophrenia and depression and those without depression**.

Characteristics		Non-SZ-MDD	SZ-MDD	*t/χ^2^*	*p*
Ages (years)		64.11 ± 3.19	64.61 ± 2.78	1.220	0.224
Education years (years)		8.37 ± 2.44	9.15 ± 2.93	2.241	**0.026**
Age of first onset (years)		30.02 ± 11.05	28.96 ± 8.32	0.770	0.442
BMI (kg/m^2^)		23.84 ± 4.41	24.25 ± 6.28	0.602	0.548
Chlorpromazine equivalent dose		205.03 (147.18)	438.33 (311.24)	2.592	**0.010**
	Positive Syndrome score	15.07 ± 5.48	16.78 ± 5.35	2.349	**0.020**
	Negative Syndrome score	23.63 ± 6.65	24.05 ± 6.91	0.463	0.643
	General Psychopathology Syndrome score	37.23 (30.07)	42.08 (36.38)	2.513	**0.011**
PANSS total score		75.41 (62.34)	82.27 (69.52)	2.334	**0.021**
HAMD total score		8.33 (5.92)	22.35 (17.64)	15.839	**<0.001**
Gender				0.125	0.724
	Male	139 (71.6%)	59 (73.8%)		
	Female	55 (28.4%)	21 (26.2%)		
Family history of mental illness				0.155	0.694
	No	174 (89.7%)	73 (91.3%)		
	Yes	20 (10.3%)	7 (8.8%)		
Diabetes mellitus				0.004	0.947
	No	139 (71.6%)	57 (71.3%)		
	Yes	55 (28.4%)	23 (28.7%)		
Hypertension				0.855	0.355
	No	144 (74.2%)	55 (68.8%)		
	Yes	50 (25.8%)	25 (31.2%)		
Cardiovascular disease				1.216	0.270
	No	181 (95.8%)	72 (92.3%)		
	Yes	8 (4.2%)	6 (7.7%)		

Note: Bold indicates *p* < 0.05.

### 3.3 Factors Associated With SZ-MDD Comorbidity

Binary logistic regression (adjusted for age, sex, antipsychotic dose) identified HAMD total score (odds ratio [OR] = 1.364, 95% CI: 1.268–1.467, *p* < 0.001), General Psychopathology Syndrome (OR = 1.064, 95% CI: 1.013–1.118, *p* = 0.012), and PANSS total score (OR = 1.062, 95% CI: 1.008–1.152, *p* = 0.002) as significant associated factors (Table 3). ROC analysis demonstrated AUCs of 0.918 for HAMD total score, 0.598 for General Psychopathology Syndrome, and 0.595 for PANSS total score (Fig. 1). The HAMD-17 total score demonstrated excellent discriminative capacity (AUC = 0.918), which is expected given the conceptual overlap between the scale and the diagnostic construct.

**Table 3.** **Associated factors of major depressive disorder in elderly patients with schizophrenia**.

Model	Unstandardized coefficient		Wald *χ^2^*	*p*	*OR*	95% confidence interval for OR		VIF	Tolerance
	*B*	Standard Error				Lower	Upper		
Education years	–0.028	0.077	0.135	0.713	0.972	0.836	1.130	1.19	0.840
Chlorpromazine equivalent dose	0.000	0.000	0.708	0.400	1.000	1.000	1.001	1.08	0.926
HAMD total score	0.310	0.037	69.657	**<0.001**	1.364	1.268	1.467	1.33	0.752
Positive Syndrome score	0.045	0.053	0.735	0.391	1.046	0.943	1.161	1.42	0.704
General Psychopathology Syndrome score	0.062	0.025	6.267	**0.012**	1.064	1.013	1.118	1.51	0.662
PANSS total score	0.060	0.019	9.972	**0.002**	1.062	1.008	1.152	1.28	0.781
Constant	–5.363	1.480	13.133	**<0.001**	0.005				

Note: Bold indicates *p* < 0.05.

**Fig. 1.**
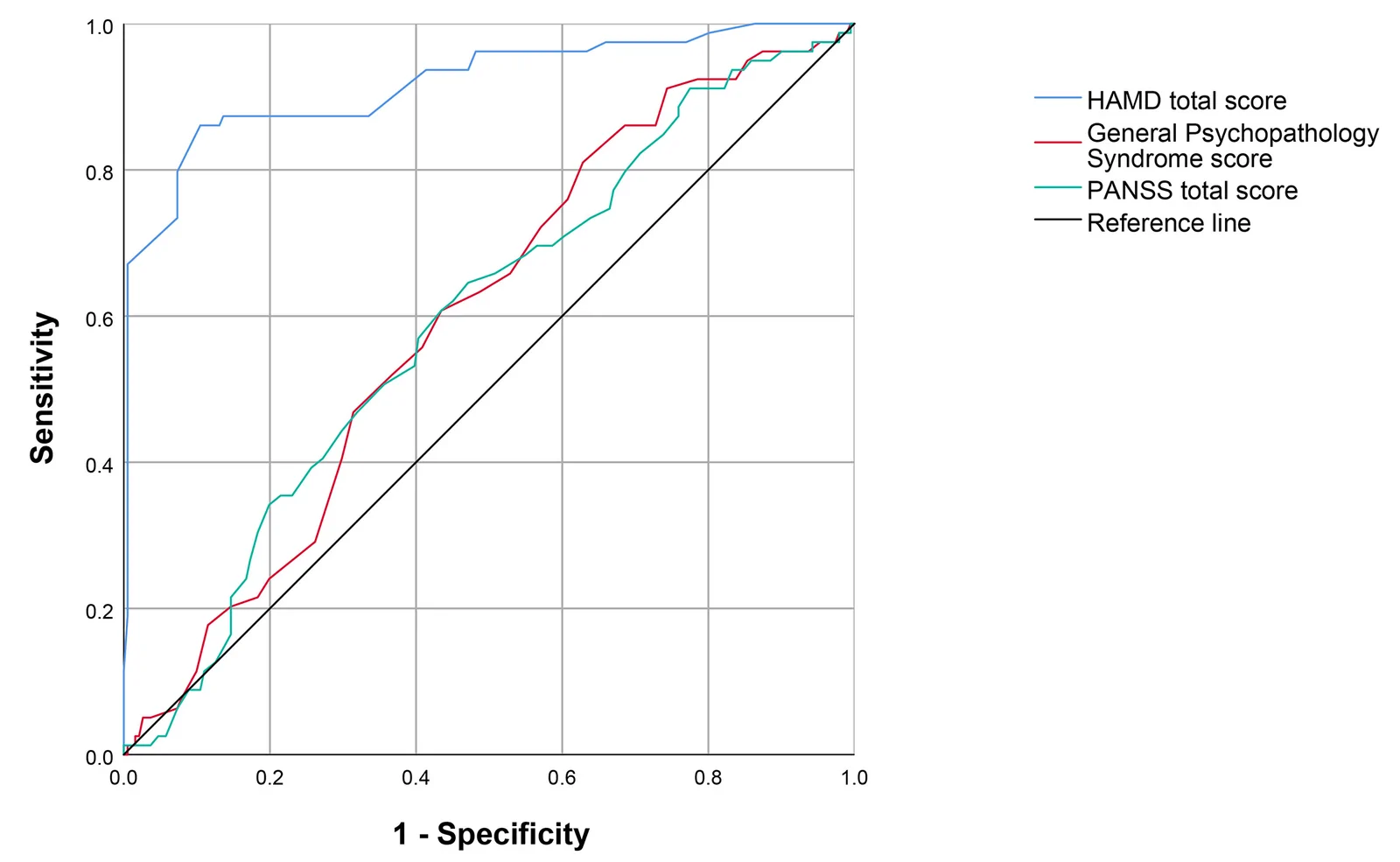
**ROC analysis of the factors influencing SZ-MDD in elderly patients with schizophrenia**. The areas beneath the curves were 0.918 for the HAMD total score, 0.598 for the General Psychopathology Syndrome score, and 0.595 for the PANSS total score, respectively.

In sensitivity analyses using generalized linear mixed models with site as a random intercept, the findings remained essentially unchanged: PANSS General Psychopathology (OR = 1.061, 95% CI: 1.010–1.115, *p* = 0.015) and PANSS Total Score (OR = 1.033, 95% CI: 1.006–1.060, *p* = 0.013) remained significantly associated with MDD comorbidity, confirming that site-level clustering did not materially affect the results.

To examine whether the association between PANSS indices and MDD comorbidity differed by gender, we conducted stratified analyses separately for male (n = 198) and female (n = 76) patients. In male patients, binary logistic regression (adjusted for age, education, and antipsychotic dosage) revealed that PANSS General Psychopathology (OR = 1.102, 95% CI: 1.018–1.193, *p* = 0.014) and PANSS Total Score (OR = 1.035, 95% CI: 1.008–1.062, *p* = 0.011) remained significant associated factors of MDD comorbidity. Similarly, in female patients, PANSS General Psychopathology (OR = 1.125, 95% CI: 1.022–1.238, *p* = 0.010) and PANSS Total Score (OR = 1.042, 95% CI: 1.010–1.075, *p* = 0.008) were also significant associated factors. No significant gender-by-PANSS interaction was detected (all *p* for interaction > 0.05), indicating that the observed associations were consistent across sexes.

### 3.4 Factors Associated With Depression Severity

Ordinal logistic regression (full sample, N = 274; depression severity categories: 0 = no depression, 1 = mild, 2 = moderate, 3 = severe) revealed that depression severity was associated with age (Wald χ^2^ = 77.49, *p* < 0.001) and PANSS total score (Wald χ^2^ = 59.34, *p* < 0.001).

### 3.5 HAMD-PANSS Score Correlations

Partial correlation analysis (adjusted for age, sex, education years, age of first-onset, Chlorpromazine equivalent dose) showed significant correlations between HAMD total score and PANSS Positive Syndrome (r = 0.233, *p* < 0.001), General Psychopathology Syndrome (r = 0.402, *p* < 0.001), and total score (r = 0.310, *p* < 0.001), all surviving Bonferroni correction (Table 4). Multivariate regression confirmed associations with General Psychopathology Syndrome (B = 0.841, t = 5.607, *p* < 0.001) and PANSS total score (B = 0.286, t = 3.377, *p* = 0.001), but not Positive Syndrome (B = 0.120, t = 1.021, *p* > 0.05).

**Table 4.** **Association between HAMD scores and PANSS scores in elderly schizophrenia patients**.

HAMD total score	Partial correlation		
	*r*	*p^a^*	*p^b^*
Positive Syndrome score	0.233	**<0.001**	**<0.001**
Negative Syndrome score	0.606	0.342	>0.05
General Psychopathology Syndrome score	0.402	**<0.001**	**<0.001**
PANSS total score	0.310	**<0.001**	**<0.001**

Note: Bold indicates *p* < 0.05. *^a ^* Adjusting for age, sex, years of education, age of first-onset, and Chlorpromazine equivalent dose. *^b ^* Bonferroni correction.

## 4. Discussion

Our study found a ​​29.2% prevalence​​ of comorbid MDD in elderly schizophrenia patients, consistent with the pooled estimate of ​​32.6%​​ reported by Etchecopar-Etchart et al. [1] in stabilized schizophrenia outpatients. However, this rate is notably lower than that in younger cohorts. For instance, Herniman et al. [23] reported ​​49.3%​​ in first-episode schizophrenia (FES) patients (mean age 21.1 years). In addition, Li et al. [8] found ​​49.3%​​ in drug-naïve FES patients (mean age 27.9 years).

Potential explanations for this discrepancy include, first, age-related neurobiological changes. Accelerated neurodegeneration in elderly schizophrenia may alter depressive symptom expression, with apathy and anhedonia potentially overlapping with negative symptoms or dementia prodromes [20,24]. Secondly, ​​our cohort excluded patients with severe somatic illnesses, potentially underestimating prevalence as chronic physical diseases increase MDD risk [1]. Finally, a diagnostic assessment was conducted using the SCID-5. This instrument shares the high specificity of the CDSS for identifying depression within schizophrenia. Previous research, however, frequently relied on the HAMD-17 scale, which demonstrates limited specificity in distinguishing depressive symptoms from the negative symptoms characteristic of schizophrenia [20,23]. The high AUC for HAMD-17, while confirming the validity of our depression assessment, does not by itself establish independent clinical screening utility beyond the scale’s inherent design; this is further discussed in the Limitations section.

​​A cardinal finding of this study is the ​​robust association between PANSS indices and comorbid MDD​​ in elderly schizophrenia patients. Specifically, both the General Psychopathology subscale and ​​the PANSS total score emerged as significant associated factors of MDD comorbidity. In contrast, HAMD-17 scores correlated strongly with both the General Psychopathology subscale and the PANSS total score. Critically, the ​​PANSS total score, a global marker of illness severity [25], demonstrated a dose-dependent relationship with depression risk, indicating that ​​elderly patients with more severe schizophrenia symptomatology face disproportionately higher MDD susceptibility​​. This result aligns with Li et al. [8], who identified PANSS general psychopathology as an MDD predictor in drug-naïve first-episode schizophrenia, reinforcing depression as an ​​independent disease dimension​​ rather than a secondary phenomenon. Mechanistically, shared neuroinflammatory pathways (e.g., interleukin-6 [IL-6]/C-reactive protein [CRP] dysregulation) may concurrently drive psychotic and depressive symptomatology [26]. In the geriatric context, this severity-depression nexus may be exacerbated by ​​age-related vulnerabilities​: diminished neuroplasticity, chronic antipsychotic exposure, and psychosocial stressors (e.g., institutionalization, disability) that amplify affective morbidity in severe schizophrenia phenotypes [27,28]. These findings underscore the need for ​​integrated severity monitoring​​ in elderly schizophrenia to identify high-risk MDD subgroups preemptively.

The relationship between negative symptoms and depression in schizophrenia has long been characterized by ​​phenomenological overlap and diagnostic ambiguity [29]​​. Seminal systematic reviews highlight that core features of depression (e.g., anhedonia, avolition, anergia) exhibit substantial convergence with negative symptoms. At the same time, affective components like low mood and suicidal ideation may retain diagnostic specificity [30]. This complexity is compounded in elderly populations, where neurodegenerative processes and chronicity may further blur symptom boundaries [31]. Contrary to expectations, our study revealed ​​no significant association between HAMD-17 scores and PANSS negative subscale scores​​ in elderly schizophrenia patients. Furthermore, we found no significant association between PANSS negative symptom scores and depression severity as measured by HAMD. This null finding aligns with Panov [32], who reported no correlation between HAMD scores and negative symptoms in treatment-resistant schizophrenia.

The absence of a significant association between negative symptoms and depressive symptoms in elderly schizophrenia patients may be attributed to several interrelated factors, each contributing to this dissociation through distinct mechanisms: ​​First​​, ​​assessment tool limitations​​ introduce diagnostic conflation. The HAMD-17 incorporates items (e.g., psychomotor retardation, loss of interest) that overlap phenotypically with core negative symptoms, thereby obscuring their discriminant validity [30,33]. Conversely, negative symptoms (e.g., blunted affect, social withdrawal) predominantly capture enduring, trait-like deficits rather than state-dependent depressive phenomena [34]. ​​Second, age-related pathophysiological changes may be associated with altered symptom expression, with neurodegenerative processes in aging potentially amplifying primary negative symptoms as more enduring deficits, while depressive features remain more closely tied to affective distress and subjective well-being. Importantly, neuroimaging evidence suggests that schizophrenia and depression share abnormalities in prediction error processing within dopamine-rich regions (e.g., striatum, prefrontal cortex), which may contribute to the overlapping yet distinct affective and cognitive manifestations observed in these conditions [35]. Third, as highlighted by Barnes and McPhillips [31], the dissociation between negative and depressive symptoms in geriatric schizophrenia may reflect the inherent difficulties in their clinical discrimination, where overlapping phenomenology and medication effects often obscure the boundaries between these domains. ​​Finally, beyond neurobiological factors, growing evidence highlights the importance of a bio‑psycho‑social framework in understanding the interplay between negative symptoms and depressive states in schizophrenia. For instance, psychological factors such as alexithymia, childhood maltreatment, and impulsivity, alongside biological markers including CRP, free triiodothyronine (FT3), and lipid profiles, have been shown to collectively predict psychological distress in schizophrenia patients [36]. In elderly patients, chronic illness progression further “decouples” these domains: negative symptoms increasingly reflect neurodegenerative disease burden, while depressive states retain links to psychosocial stressors independent of symptom severity. ​​Thus, these factors, including methodological constraints, neurobiological aging, and functional divergence, highlight the need for age-adapted assessment frameworks to disentangle depression from negative symptoms in geriatric schizophrenia.

​​Moreover, we observed that ​​patients with comorbid MDD received significantly higher antipsychotic dosage​​ (as chlorpromazine equivalents) compared to those without MDD. This finding stands in contrast to Fond et al. [26], who reported ​​no association between antipsychotic type/dosage and depression remission​​. The discrepancy may be attributed to ​​geriatric-specific pharmacokinetic profiles​​: age-related metabolic alterations heighten susceptibility to drug side effects (e.g., sedation, Parkinsonism) [37] that mimic or exacerbate depressive symptomatology. Furthermore, the ​​prolonged illness duration​​ in our cohort (mean onset age: 29.7 years) potentially reflects treatment resistance linked to severe psychopathology, wherein higher antipsychotic dosing becomes clinically necessary [38].

Critically, while antipsychotics themselves ​​may contribute to both negative and depressive symptoms​​ in elderly schizophrenia, our adjusted analyses demonstrated that ​​SZ-MDD associations with PANSS total scores​​ and general psychopathology​​ ​​persisted after controlling for antipsychotic dosage​​. This result indicates that psychopathological severity, rather than medication effects, constitutes the primary driver of MDD comorbidity [8,39,40]. Future investigations should prioritize ​​recruiting antipsychotic-naïve elderly schizophrenia patients​​ to elucidate baseline MDD prevalence​​ unconfounded by iatrogenic effects.

There is a growing appreciation of the role of early developmental processes in the pathoetiology of both depression and schizophrenia [41], primarily driven by an array of developmental stressors/trauma [42]. Recent work indicates that developmental stressors/trauma act via increased cortisol effects at the glucocorticoid receptor in astrocytes to epigenetically modulate astrocyte mitochondrial function [43,44], leading to an increased susceptibility to symptom exacerbation by subsequent stressors, as in other psychiatric conditions [45]. Future research should clarify whether the correlations of HAMD-17 and PANSS have their physiological roots in developmental processes and how these interact with the wider physiological changes occurring over the course of aging [46]. The dramatic loss of melatonin over the course of aging [47] can be exacerbated in neuropsychiatric conditions leading to alterations in circadian dampening and resetting at night, partly by the loss of melatonin’s suppression of the glucocorticoid receptor nuclear translocation [48], changing how central nervous system (CNS) and systemic processes are prepared for the coming day [49] primarily via impacts on mitochondrial function [50].

### Limitations

​​​​This study is subject to several methodological constraints, which warrant cautious interpretation of the findings. ​​First​​, the cross-sectional design inherently precludes causal inferences regarding the temporal and mechanistic relationships between MDD and schizophrenia. ​​Second, the exclusive inclusion of a single ethnic cohort (Chinese participants) limits generalizability to diverse populations; genetic polymorphisms (e.g., brain-derived neurotrophic factor [BDNF] Val66Met) and culturally mediated factors (e.g., illness attribution models) may confound observed associations, as evidenced in prior research [8]. Additionally, all participants were inpatients, which may limit the generalizability of our findings to outpatient or community‑dwelling elderly populations with schizophrenia. Future studies should include diverse settings to enhance external validity. Third​​, assessment tool bias remains a critical concern: the HAMD-17’s propensity to conflate negative symptoms (e.g., psychomotor retardation) with core depressive features likely inflates MDD prevalence estimates, whereas instruments like the CDSS or structured clinical interviews (e.g., SCID) would enhance nosological specificity. ​​Fourth​​, selection bias arises from the systematic exclusion of patients with severe comorbidities (e.g., dementia, advanced cardiovascular disease), potentially underestimating true MDD prevalence in frail elderly subgroups who face compounded neuropsychiatric burdens. Fifth, the ROC analysis for HAMD-17, while demonstrating excellent discriminative capacity (AUC = 0.918), should be interpreted with caution. As HAMD-17 is a direct measure of depressive symptom severity, its high AUC against an MDD diagnosis determined by SCID-5 is inherently expected and does not by itself establish independent clinical screening utility. This analysis was included primarily as a benchmark to validate the robustness of our depression ascertainment and to contextualize the discriminative performance of the PANSS indices, which showed only modest AUC values (≈0.60). Sixth, several potentially important geriatric and medication‑related confounders were not assessed in this study. While we adjusted for core covariates (age, sex, education, BMI, antipsychotic dosage, and chronic somatic conditions), we did not collect data on cognitive function, antidepressant use, or functional disability. These unmeasured factors may have contributed to residual confounding, particularly in an elderly population where cognitive decline, social isolation, and medication burden are prevalent. Finally​​, the absence of biomarker data—particularly inflammatory markers (e.g., IL-6, CRP) and neurotrophic factors (e.g., BDNF, glial cell line-derived neurotrophic factor [GDNF]) [39]—restricts mechanistic insights into pathways linking psychotic symptomatology, neuroinflammation, and affective morbidity in late-life schizophrenia.

## 5. Conclusion​​s

In this multicenter sample of elderly Chinese inpatients with schizophrenia, comorbid MDD was present in 29.2% of participants, with PANSS total score and General Psychopathology subscale identified as significant correlates of MDD comorbidity. However, given the modest discriminative performance of PANSS indices (AUC ≈ 0.60), their role in clinical practice should be viewed as adjunctive to comprehensive psychiatric evaluation rather than as a standalone screening tool.

## Data Availability

The data supporting this study’s findings are available on request from the corresponding authors.
